# Rain-Shelter Cultivation Modifies Carbon Allocation in the Polyphenolic and Volatile Metabolism of *Vitis vinifera* L. Chardonnay Grapes

**DOI:** 10.1371/journal.pone.0156117

**Published:** 2016-05-24

**Authors:** Yuan Gao, Xiao-Xi Li, Mei-Mei Han, Xiao-Fan Yang, Zheng Li, Jun Wang, Qiu-Hong Pan

**Affiliations:** 1 Center for Viticulture and Enology, College of Food Science and Nutritional Engineering, China Agricultural University, Beijing, China; 2 Food Science and Human Nutrition Department, Institute of Food and Agricultural Sciences, University of Florida, Gainesville, Florida, United States of America; South China Agricultural University, CHINA

## Abstract

This study investigated the effect of rain-shelter cultivation on the biosynthesis of flavonoids and volatiles in grapes, with an aim of determining whether rain-shelter application could help to improve the sensory attributes and quality of grapes. *Vitis vinifera* L. Chardonnay grapes, grown in the Huaizhuo basin region of northern China, were selected within two consecutive years. A rain-shelter roof was constructed using a colorless polyethylene (PE) film with a light transmittance of 80%. Results showed that rain-shelter treatment did not affect the accumulation of soluble solids during grape maturation. However, the allocation of assimilated carbon in phenolic and volatile biosynthetic pathways varied significantly, leading to alterations in polyphenolic and volatile profiles. The rain-shelter cultivation enhanced the concentration of flavan-3-ols via the flavonoid-3’5’-hydroxylase (F3’5’H) pathway, but reduced the level of flavonols and flavan-3-ols via the flavonoid-3’-hydroxylase (F3’H) pathway. In addition, the rain-shelter cultivation significantly enhanced the synthesis of fatty acid-derived volatiles, isoprene-derived terpenoids and amino acid-derived branched-chain aliphatics, but led to a decrease in the accumulation of isoprene-derived norisoprenoids and amino acid-derived benzenoids. Principal component analysis revealed some key compounds that differentiated the grapes cultivated under open-field and rain-shelter conditions. Moreover, the effect of the rain-shelter application on the accumulation of these compounds appeared to be vintage dependent. The alteration of their profiles caused by the rain-shelter treatment was significant in the vintage that received higher rainfall, which usually took place in the first rapid growth and veraison phases.

## Introduction

Rain-shelter cultivation is an important cultivation approach that has been widely applied to grapes in rainy regions. Transparent plastic films are normally used to cover the roof and lateral belts of vineyards during grape development stages, preventing rainfall damage and eliminating fruit disease incidences [1]. Grape rain-shelter cultivation was introduced to the southern and eastern coastal regions of China in the 1980s from western Japan. In these regions of China, the annual rainfall ranges from 1,500 to 3,000 mm, and the rainy season usually occurs from June to September. Excessive rainfall during grape growing season results in heavy shattering and serious diseases, such as anthracnose and downy mildew. Numerous studies have confirmed that simple rain-shelter cultivation can effectively reduce these disease problems [2, 3]. Rain-shelter cultivation also changes the microclimates of vineyards due to the application of plastic films. It has been reported that solar radiation and photosynthetically active radiation were significantly reduced in grapevines covered with plastic films [4, 5]. Meanwhile, rain-shelter cultivation helps to increase the air temperature surrounding grapes and reduces diurnal air relative humidity [4]. Microclimate variation caused by rain-shelter application affects grape quality. For example, it has been reported that grapes cultivated under rain shelter had higher levels of sugar and soluble solids compared with those grown under open-field cultivation, and rain-shelter application delayed the maturation of grapes [6, 7].

Phenolic and volatile compounds are important secondary metabolites synthesized in grapes during grape development period, and they significantly contribute to the organoleptic features of grapes and wines. In particular, phenolic compounds play primary roles in the color, taste and astringency, whereas wine flavor and aroma is essentially determined by the volatile profile [8, 9]. Regarding their biosynthetic pathways, phenolic compounds in grape berries are produced through phenylpropanoid-flavonoid metabolism that begins with phenylalanine, whereas volatile compounds are synthesized via multiple pathways that starts from isoprenes, amino acids, and polyunsaturated fatty acids [10, 11, 12].

Rain-shelter cultivation has been reported to affect the accumulation of phenolic and volatile compounds in grapes by regulating the expression of biosynthetic genes [7, 13, 14]. As a result, the profiles (composition and/or distribution) of phenolic and volatile compounds were altered [3, 15]. However, these previous studies were merely concerned on some specific phenolic and/or volatile compounds. The evolution of both phenolic and volatile patterns during grape growing season under rain-shelter approach has not been well investigated. In particular, assimilated carbon flow and allocation in important flavor and aroma metabolisms in grapes under rain-shelter cultivation have not been well understood. Therefore, we adopted rain-shelter technique to Chardonnay (*Vitis vinifera* L.) grapes and compared the evolution of both phenolic and volatile metabolites with those under open-field cultivation during grape development stages. The variation in the phenolic and volatile profiles was explained through their metabolic pathways. This work could provide an integral understanding of the evolution of the main phenolic and volatile metabolisms and their association during berry development. The research findings could also help to evaluate the potential of rain-shelter application in wine grape production, and offer further references for a good balance of flavor and aroma in grapes.

## Materials and Methods

### Experimental design

The experiment was carried out in a commercial Chardonnay (*Vitis vinifera*, L.) vineyard in Chateau Changyu Afip Global (44°30′N, 116°80′E), Miyun County, Beijing, China, during the grape-growing season over a two-year period (2012 and 2013). We confirm that the vineyard owner gave us permission to conduct the study. The land accessed is privately owned and no protected species were sampled. (The name of the manger is Zhan-Wei Yan, e-mail: yanzhanwei0104@hotmail.com.) This vineyard covers an area of approximate 100 hectares and is located in a plain field with sandy soil. The own-rooted grapevines were planted in 2007 in a north-south row orientation with an intra-row spacing of 2.5 m and an intra-shoot spacing of 1.0 m. These vines were trained to form a vertical shoot positioning training system with a sloping trunk and unilateral cordon 0.8 m above ground. They were spur-pruned annually and covered with soil to ensure overwinter protection. The shoots were positioned vertically upright with the aid of wires and each vine carried *ca*. 20 grape clusters. Experimental rows were selected from the 20 central rows of this vineyard. To ensure the effect of the rain shelter, we selected 10 rows on the eastern side for the rain-shelter treatment, and the remaining 10 rows were used for the open-field treatment (the control). In both of the treatments, three sampling units were artificially divided to create three biological replicates (50 vines per replicate). All of the experimental units were subjected to similar viticultural management, including pest and disease control, fertilization, and canopy management.

**Fig 1:**
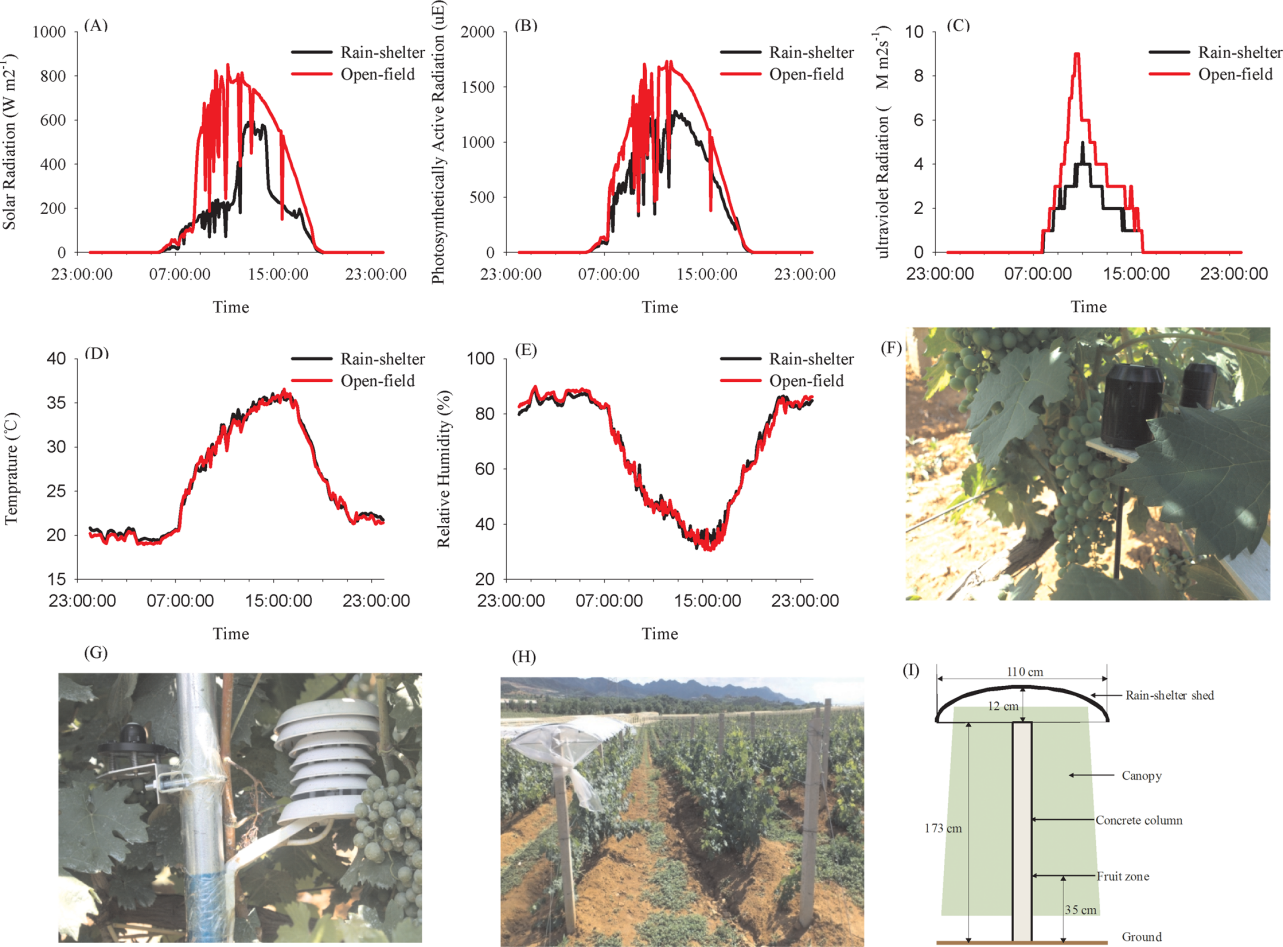
Main meteorological parameters, collectors and rain-sheltered parameters. Main meteorological parameters around the cluster under rain-shelter and open-field cultivation (A, B, C, D, E), meteorological data collector (F, G), rain-shelter shed and related parameters (H, I).

A rain-shelter roof was constructed using a colorless polyethylene (PE) film with a thickness of 0.10–0.12 mm and an 80% light transmittance. For each row, the rain-shelter frame had a 1.85 m height from the ground, a 1.1 m width, and a 1.25 m arc length. The frame was designed as a jack-roof to eliminate rain and provide natural ventilation (**Fig 1H & 1I**). The rain-shelter roof was set up on the 14^th^ day after flowering (DAF, 14^th^ June) in 2012 and 36 DAF (13^th^ July) in 2013, respectively.

To understand the change in microclimate around the grapevines under the rain shelter and the open-field treatments, data collectors and testing probes were installed around the clusters (**Fig 1F & 1G**). Temperature, relative air humidity, solar radiation, photosynthetically active radiation, and ultraviolet radiation were recorded every 5 minutes using a HOBO Remote Monitoring System (Onset, Bourne, MA, USA). The meteorological data were collected from 7 DAF until harvesting. **Fig 1A–1E**shows an example of the daily variation in these meteorological parameters on August 24, 2013. It was observed that more than 20% of the solar radiation and the photosynthetically active radiation reaching the grape clusters were removed. Ultraviolet radiation was reduced by 40% around the rain-shelter cultivated grape clusters. No significant differences in temperature or relative humidity were observed between these two cultivation treatments.

### Sample collection

Chardonnay grapes in this region were approximately harvested at 100 DAF. The grapes were sampled at 20, 37, 64, 71, 78, 87 and 97 DAF for the 2012 vintage, and 14, 28, 41, 57, 67, 78 and 97 DAF for the 2013 vintage. For each replicate, about 600 berries were randomly collected from 300 clusters on each sampling date. Two or three healthy berries with a pedicel of approximate 2 mm were collected from one cluster. The sampling time was fixed at 8:00–10:00 in the morning. The harvested samples were placed in plastic bags and transported on ice to the laboratory (about one hour by car). From these samples, 200 fresh berries per replicate were used for the determination of physicochemical indices, including berry weight, volume, Brix, pH, and titratable acid. The remaining samples were immediately frozen in liquid nitrogen and stored at -80°C. When all sampling for one grape-growing season was completed, approximately 150 grape berries per replicate from each sampling date were peeled, ground under liquid nitrogen, and lyophilized for 24 h at -50°C using an LGJ-10 vacuum freeze-dryer (Vibra-Schultheis, Offenbach am Main, Germany). The resultant freeze-dried skin powders were stored at -40°C for the analysis of flavonols and flavan-3-ols.

### Determination of physicochemical parameters

Briefly, 100 fresh berries of each replicate were placed into a measuring cylinder to measure the volume per 100 berries. Meanwhile, 50 berries were squeezed to obtain the juice using a hand juice crusher. The soluble solids content (SSC, Brix) of the juice was measured using a digital hand-held refractometer (PAL-2, ATAGO, Tokyo, Japan), and the pH value was determined using an electronic pH meter (FE20, Mettler Toledo, Greifensee, Switzerland). After the juice was diluted 5-fold using deionized water, the titratable acidity was measured by titrating the diluted juice to pH 8.2 using 0.1 M NaOH. The titratable acidity was expressed as g L^-1^ of tartaric acid.

### Phenolic compound extraction and analysis

#### Phenolic acid extraction and analysis

Phenolic acids generally exist in tartaric ester form in grape hypodermal cells and the mesocarp and placental cells of grape pulp [16]. The seeds were removed from the grape berries, and the extraction was performed in an alkaline solution to release phenolic acids from their ester forms. Fifty grape berries (without seeds) per replicate were ground into powder under liquid nitrogen, and the powder was divided into two subsamples. One subsample (5.0 g) was transferred to a 50-mL tube and mixed with 25 mL of extraction solution consisting of 4 mol L^-1^ NaOH, 1% ascorbic acid, and 10 mmol L^-1^ ethylenediamine tetraacetic acid (EDTA). The head space of the tube was filled with nitrogen to protect against oxidation, and then the sealed tube was sonicated for 3 min, followed by 8 h shaking at 35°C. Then, the resultant mixture was adjusted to pH 2.0 using 1 mol L^-1^ HCl, and then centrifuged at 8,000 × *g* for 20 min. The supernatant was collected and mixed with 30 mL diethyl ether. The organic phase was collected and the aqueous phase was re-extracted four more times using the same volume of diethyl ether. All the organic phases were pooled and then concentrated to dryness in a rotary evaporator (SY-2000, Shanghai Yarong Biochemistry Factory, Shanghai, China) at 35°C. The residues was re-dissolved in 500 μL of methanol, and analyzed using a high performance liquid chromatography (HPLC) mass spectrometer (MS) after filtration through 0.45-μm membrane filters.

A reverse phase C18 column Zorbax SB (250 mm×4.0 mm i.d., 5 μm particle size) was used for the separation of phenolic acids on an Agilent 1100 series LC-MSD trap VL system (Agilent Technologies, Santa Clara, CA, USA). The mobile phases consisted of (A) methanol/acetic acid/water (10/2/88, v/v/v) and (B) methanol/acetic acid/water (90/1.5/8.5, v/v/v). The gradient program was performed as follows: from 0 to 3.6% B for 7 min, from 3.6% to 15% B for 19 min, from 15% to 25.5% B for 6 min, from 25.5% to 29.7% B for 3 min, from 29.7% to 45.5% B for 10 min, and from 45.5% to 0% B for 8 min. The flow rate was 1 mL min^-1^ and the injection volume was 10 μL. The column was maintained at 30°C. The detection wavelength was set at 325 nm for gentisic acid, chlorogenic acid, caffeic acid, and salicylic acid. The other phenolic acids were detected at 275 nm. The identification of phenolic acids was confirmed by spectral analysis and retention time with standards, and their concentrations were calculated based on the corresponding standard curves.

#### Flavonol extraction and analysis

Flavonols were extracted from the grape skin according to a published method with minor modifications [17]. The dry skin powder (0.5 g) was mixed with 15 mL of 50% ethanol containing 1% acetic acid. The resultant mixture was sonicated for 35 min and then centrifuged at 8,000 *× g* for 10 min. The supernatant was collected. The above extraction procedure was repeated four times. The combined supernatant was mixed with 50 mL of distilled water and then extracted with 40 mL ethyl acetate. This extraction was repeated three times. All the organic phases were pooled and then evaporated to dryness at 30°C using a rotary evaporator (SY-2000, Shanghai Yarong Biochemistry Factory, Shanghai, China). The resultant dry residue was re-dissolved in 2 mL of 25% methanol/ultrapure water (v/v), and then filtered through 0.22-μm nylon membranes before LC-MS analysis.

A Bruker amaZon SL series LC-UV-MS was equipped with a reverse phase column (Zorbax Eclipse XDB-C18, 4.6 × 250 mm, 5 μm) for the flavonol analysis. The column temperature was maintained at 40°C, and the injection volume was 50 μL. The mobile phase was comprised of (A) acetonitrile/water/formic acid (5/86.5/8.5, v/v/v) and (B) acetonitrile/methanol/water/formic acid (25/45/21.5/8.5, v/v/v/v). The gradient program was as follows: 0–7 min, 0% B; 7–24.2 min, 0–14.2% B; 24.2–27 min, 14.2%-15.7% B; 27–27.4 min, 15.7%-16.3% B; 27.4–33.4 min, 16.3%-18.8% B; 33.4–39 min, 18.8%-23.5% B; 39–45 min, 23.5%-26% B; 45–47 min, 26%-27.4% B; 47–51.6 min, 27.4%-32% B; 54–55.2 min, 33.4%-34.6% B; 55.2–58.2 min, 34.6%-36.4% B; 58.2–61.8 min, 36.4%-40% B; 61.8–62.3 min, 40%-60% B; 62.3–67.8 min, 60%-100% B; and 67.8–78.4 min, 100%-0% B. The flow rate was set at 1 mL min^-1^, and flavonols were detected at 360 nm. The mass spectrometry (MS) conditions were set as follows: negative electrospray ionization (ESI) interface, 30 psi nebulizer pressure, 10 mL min^-1^ dry gas flow rate, 325°C dry gas temperature, and all mass scan mode from *m/z* 0 to 1000. The identification of flavonols was confirmed by retention time, mass spectra, and MS library according to the available standards. These compounds were quantified as quercetin-3-*O*-glucoside equivalents.

#### Flavan-3-ol extraction and analysis

Falvan-3-ols were also extracted from the grape skins. To determine the total concentration of various flavan-3-ol units, we conducted acid cleavage in the presence of excess phloroglucinol to obtain the component units of their oligomers and polymers [18]. The extraction procedure and HPLC-MS analysis of flavan-3-ols were carried out according to a published method [19]. Briefly, the grape skin powder (0.1 g) was mixed with 1 mL of phloroglucinol in a 10-mL centrifuge tube. The mixture was then placed in a water bath (YLE-1000, Beijing Changfeng Instrument Company, Beijing, China) at 50°C for 20 min. Afterwards, the resultant extract was mixed with 1 mL of sodium acetate solution, and then centrifuged at 10,000 × *g* for 10 min to collect the supernatant. This extraction was repeated three times. Subsequently, all the extracts were combined and filtered through 0.22-μm organic membranes prior to HPLC-MS analysis.

Flavan-3-ol analysis was performed using a Bruker amaZon SL series LC-UV-MS equipped with a reverse phase column (Zorbax Eclipse XDB-C18, 4.6 × 250 mm, 5 μm). The injection volume was 25 μL. A gradient consisting of (A) acetic acid/water (2/998, v/v) and (B) acetonitrile/solvent A (4/1, v/v) was applied with a flow rate of 1 mL min^-1^. The gradient program was as follows: 0–10% B for 20 min, 10%-15% B for 10 min, 15%-20% B for 10 min, 20%-33% B for 10 min, 33%-40% B for 5 min, 40%-100% B for 3 min, 100% B for 5 min, and 100%-10% B for 11 min. The column temperature was 25°C and the detection wavelength was 280 nm. The mass spectrometry (MS) conditions were as follows: negative electrospray ionization (ESI) interface, 30 psi nebulizer pressure, 10 mL min^-1^ dry gas flow rate, 325°C dry gas temperature, and scanned from *m/z* 100 to 1500 by using all mass scan mode. The identification for flavan-3-ols was determined by the comparison of their mass spectra and retention time with the available standards. The terminal subunits (flavan-3-ol monomers) and extension subunits (electrophilic flavan-3-ol intermediates) released from proanthocyanidins were also identified by mass spectrometry information and retention time. The quantification of flavonol components (including extension units) was based on their respective standards.

### Volatile compound extraction and analysis

For each sample, 200 grape berries were de-seeded and ground under liquid nitrogen, and then the flesh was mashed and blended. After cold maceration for 120 min, the flesh was immediately centrifuged at 6,000 *× g* at 4°C for 10 min to obtain clear grape juice. The juice was divided into two subsamples for the extraction of free and glycosidically bound volatiles.

#### Free volatile extraction

For each subsample, 5 mL of the juice, 1.00 g of NaCl, and 10 μL of 4-methyl-2-pentanol (internal standard, 1.0018 g L^-1^) were mixed in a 15-mL airtight vial containing a magnetic stirrer. Head space-solid phase microextraction (HS-SPME) was used for free volatile compound extraction according to our previous studies [20]. In brief, the vial containing the sample was equilibrated at 40°C for 30 min with agitation. Then, the pretreated SPME fiber (50/30-μm DVB/Carboxen/PDMS, Supelco, Bellefonte, PA., USA) was inserted into the headspace for 30 min. Afterwards, the fiber was instantly desorbed in the GC injector for 8 min.

#### Bound volatile extraction and isolation

Bound volatile extraction followed our previous study [21]. Briefly, 2 mL of the grape juice in each subsample was eluted through a Cleanert PEP-SPE cartridge column (200 mg/6 mL; Bonna-Agela Technologies, Tianjin, China) preconditioned using 10 mL of water and then 10 mL of methanol. Sugars, acids, and other polar compounds were initially removed from the column using 5 mL of water. Then, the free volatiles were eluted from the column using 5 mL of dichloromethane. Eventually, the bound volatiles were eluted from the column using 20 mL of methanol with a flow rate of 2 mL min^-1^, and then evaporated to dryness under vacuum at 30°C. The dry bound volatiles were then re-dissolved in 5 mL of 2 M citric/phosphate buffer (pH 5.0). Subsequently, the bound volatiles were released with AR2000 (100 μL, 100 mg L^-1^ in 2 M citrate/phosphate buffer at pH 5.0) for 16 h at 40°C. The released bound volatiles were collected through the same SPME method as the free volatiles.

#### GC-MS analysis

Volatile analyses were performed using an Agilent 7890 GC system equipped with a quadrupole mass spectrometer Agilent 5975C (Agilent, Santa Clara, CA, USA). A 60 m × 0.25 mm id HP-INNOWAX capillary column with a 0.25-μm film thickness (J&W Scientific, Folsom, CA, USA) was used for the separation of volatiles. The GC conditions were based on our previous research [20]. The samples were injected in a splitless mode, and helium (purity>99.999%) was used as the carrier gas at 1 mL min^-1^. The operation conditions were as follows: GC injector temperature, 250°C; electron impact (EI) mode, ionization energy, 70 eV; source temperature, 230°C; interface, 280°C; and *m/z* 20–350 full-scan mode. The temperature program started at 50°C (held for 1 min), and then increased to 220°C using a rate of 3°C min^-1^ (held for 5 min).

Retention indices were calculated after analyzing C6-C24 n-alkane series (Supelco, Bellefonte, PA, USA) under the same chromatographic conditions. The identifications were based on the retention indices of reference standards in our laboratory and mass spectra in the standard NIST08 library. When reference standards were not available, volatile compounds were tentatively identified by comparing mass spectra with the standard NIST08 library and a comparison of retention indices sourced in the literature.

Quantification was carried out according to the internal standard curve method, using 4-methyl-2-pentanol as the internal standard [20]. According to the average concentration of sugar and acids in grape juice, a synthetic matrix was prepared using distilled water with 1% (v/v) ethanol containing 200 g L^-1^ glucose and 7 g L^-1^ tartaric acid, and the pH was adjusted to 4.3 using 1 M NaOH solution. Each standard was dissolved with ethanol (HPLC quality) and combined together. Then, all of the standard stock solutions were dissolved in the synthetic matrix at the concentrations found in grape juice, and then diluted into fifteen levels in succession. The aroma standards of each level were extracted and analyzed under the same condition as the grape samples. In addition, volatile compounds without reference standards were estimated using those standards that had the same functional group and/or similar numbers of carbon atoms.

### Statistical analyses

Comparison of means was analyzed using an independent-sample t test with a significance level of *p* <0.05. Two-way analysis of variance and principal component analysis (PCA) were performed using SPSS 20.0 statistical package for Windows (SPSS Inc., Chicago, IL, USA). Two-way analysis of variance was performed to evaluate the effects of vintage and cultivation on various polyphenols and volatiles in grape berries at significance levels of *p* <0.05 and *p* <0.01. Sigma Plot 10.0 (Systat Software Inc., Richmond, CA, USA) was used to construct the graphs.

## Results

### Physicochemical parameters

**Fig 2:**
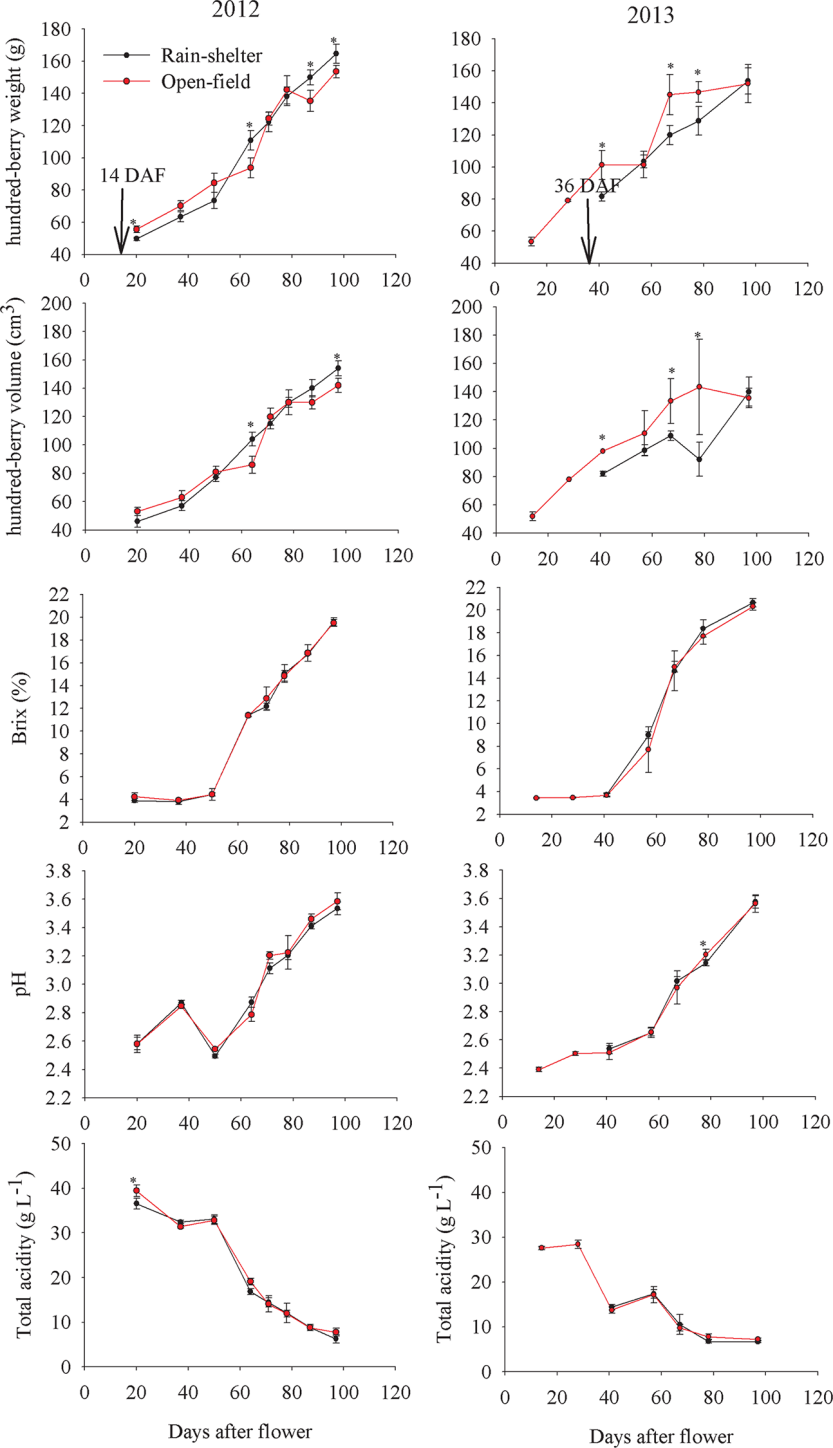
The basic physical and chemical indicators in grapes under rain-shelter and open-field cultivation. Including hundred-berry weight, hundred-berry volume, Brix, pH and total acidity in the vintage of 2012 and 2013. The signal “*” for the same compound indicates a significant difference between the rain-shelter and open-field cultivation (*p*<0.05). The ‘arrow’ indicates the time that the rain-shelter shed was implemented.

The rain-shelter treatment, compared with the open-field cultivation, resulted in a reduction of the 100-berry weight in the early phase of the berry development (pre-veraison) but a significant increase in the berry maturation phase in the 2012 vintage. In the 2013 vintage, the 100-berry weight under the open-field cultivation was higher than that under the rain-shelter cultivation. However, the berry weight of these cultivated grapes did not show significant differences at the harvest point. Moreover, the rain-shelter cultivation did not alter the soluble solids content or the titratable acidity level in either the 2012 or the 2013 vintage (**Fig 2**).

### Effect of rain-shelter cultivation on phenolic accumulation

#### Phenolic acids

**Fig 3:**
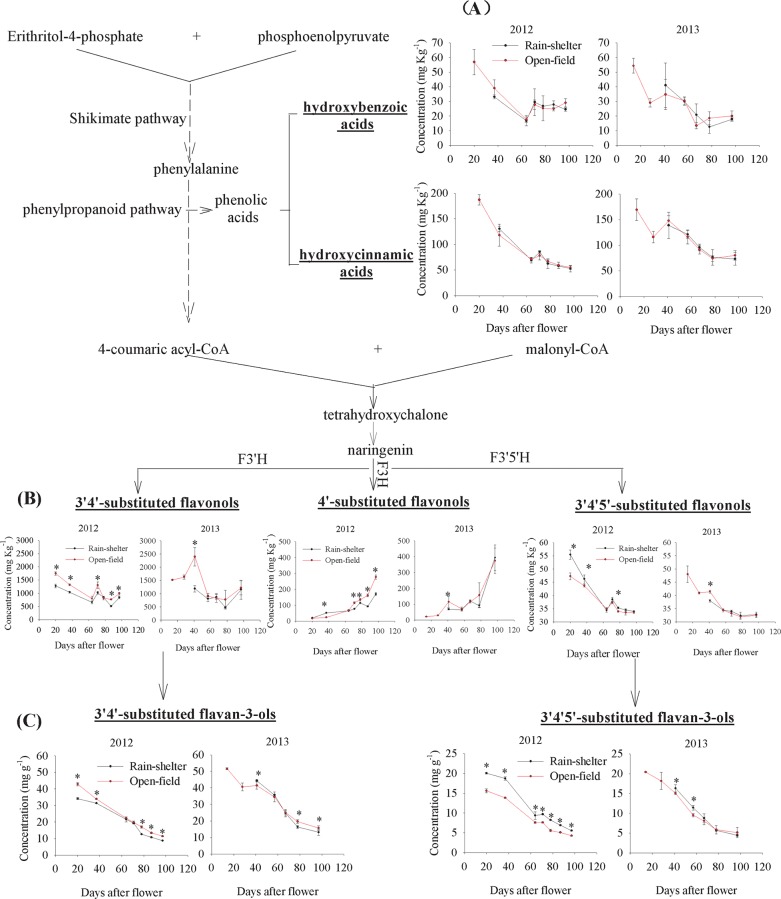
Total concentrations of phenolic acids, flavonols, and flavan-3-ols in grapes under the two treatments. (A), (B), and (C) represents the concentrations of phenolic acids (hydroxybenzoic acids and hydroxycinnamic acids), various substituted flavonols, and flavan-3-ols in grape berries, respectively, under rain-shelter and open-field cultivation in 2012 and 2013. F3H: flavonoid 3-hydroxylase; F3’H: flavonoid 3’-hydroxylase; F3’5’H: flavonoid 3’5’-hydroxylase. * represents a significant difference in the concentrations between the two types of cultivation (*p*<0.05).

Phenolic acids are the products of phenylpropanoid metabolism that is initiated with phenylalanine, a product of the shikimate pathway [22]. The total phenolic acid concentration gradually decreased during the grape maturation process. However, no significant differences were observed in the total phenolic acid concentration of these two cultivated grapes in either vintage (**S1A Fig**). According to their structural features, phenolic acids are divided into hydroxybenzoic and hydroxycinnamic acids. The rain-shelter cultivation did not significantly change the production efficiency of the hydroxbenzoic or hydroxycinnamic acid in either vintage (**Fig 3A**). With respect to individual phenolic acids, eight phenolic acids were detected in the grape samples, including four hydroxybenzoic acids (gallic acid, protocatechuic acid, *p*-hydroxybenzoic acid, and syringic acid) and four hydroxycinnamic acids (chlorogenic acid, caffeic acid, *p*-coumaric acid, and ferulic acid). During the grape maturation, these compounds, except for protocatechuic acid, showed a decreasing trend. Of these, gallic acid, protocatechuic acid, *p*-coumaric acid, and caffeic acid accounted for more than 90% of the total concentration at the harvest point of both of the vintages (**Table 1**). The rain-shelter cultivation did not significantly impact the metabolism of the phenolic acids although the ferulic acid level was lower in the rain-shelter cultivated grapes at the harvest point of the 2012 vintage.

**Table 1:** Concentrations of various polyphenols in the commercially harvested grape berries under rain-shelter and open-field cultivation in the 2012 and 2013 vintages (mg Kg^-1^).

Numbers	Compounds	2012		2013	
		Rain-shelter	Open-field	Rain-shelter	Open-field
**Phenolic acid compounds**					
A1	Gallic acid	11.75±1.48	13.58±1.92	11.72±1.33	14.59±3.31
A2	Protocatechuic acid	11.28±2.11	13.8±1.81	5.28±0.81	4.62±0.64
A3	p-Hydroxybenzoic acid	1.47±0.44	1.63±0.73	0.28±0.17	0.1±0.03
A4	Chlorogenic acid	0.63±0.35	0.95±0.21	0.67±0.39	0.72±0.13
A5	Caffeic aci	41.8±4.36	42.96±2.7	54.35±9.67	59.34±6.9
A6	Syringic acid	tr	0.11±0.01	0.73±0.41	0.71±0.1
A7	p-Coumaric acid	8.14±1.22	7.83±0.45	16.53±3.96	18.24±2.41
A8	Ferulic acid	**1.99±0.17**	**3.15±0.41**	1.61±0.23	1.72±0.17
**Flavonol compounds**					
A9	Dihydroquercetin-3-O-glucoside	15.18±0.42	15.46±0.46	14.88±0.02	14.93±0.05
A10	Myricetin-3-O-glucuronide	**16.72±0.07**	**1****5.65±0.09**	15.19±0.18	15.02±0.16
A11	Myricetin-3-O-glucoside	**17.3±0.26**	**18.00±0.32**	17.56±0.75	17.37±0.48
A12	Quercetin-3-O-galactoside	**39.12±1.04**	**64.45±1.58**	100.02±26.85	93.97±14.07
A13	Quercetin-3-O-glucuronide	**358.17±20.58**	**301.33±15.58**	296.42±128.64	365.30±102.85
A14	Quercetin-3-O-glucoside	**389.76±19.87**	**570.02±34.78**	701.77±219.63	709.67±148.79
A15	Kaempferol-3-O-galactoside	**35.73±1.68**	**55.25±0.68**	77.36±27.86	72.82±14.52
A16	Kaempferol-3-O-glucuronide	**31.12±1.12**	**41.44±1.55**	47.35±12.63	50.12±9.65
A17	Kaempferol-3-O-glucoside	**102.83±6.41**	**180.68±11.38**	268.99±110.13	250.31±56.25
A18	Isorhamnetin-3-O-glucoside	20.40±0.19	20.49±0.23	20.82±0.77	21.69±1.52
A19	Quercetin	15.64±0.13	15.27±0.27	15.35±0.39	15.15±0.23
**Flavan-3-ol compounds**					
A20	(+)-Catechin	**0.90±0.03**	**1.16±0.02**	1.33±0.33	1.62±0.31
A21	(-)-Epicatechin	**5.79±0.07**	**7.09±0.11**	**10.57±1.7**	**12.53±0.96**
A22	(-)-Epigallocatechin	**5.55±0.15**	**4.29±0.07**	4.45±0.3	5.16±1.4
A23	(-)-Epicatechin-3-O-gallate	**1.98±0.06**	**3.18±0.04**	**1.24±0.15**	**1.53±0.13**

Bold & underline represents that the compund concentration has statistically-significant difference between the rain-shelter and open-field grapes in the same vintage (p<0.05)

#### Flavonols

Flavonols are classified into quercetin-, kaempferol-, and myricetin-type flavonols according to their biosynthetic pathways. These three types of flavonols are B-ring 3’4’-substituted, 4’-substituted, and 3’4’ 5’-substituted flavonols biosynthesized with the activity of flavonoid-3’-hydroxylase (F3’H), flavonoid-3-hydroxylase (F3H), and flavonoid-3’5’-hydroxylase (F3’5’H), respectively (**Fig 3B**). The total flavonol concentration showed a “W” tendency during the grape maturation of the two vintages (**S1B Fig**). Significant differences in the total flavonol concentration were observed in the grapes under the two cultivation modes at various sampling points in 2012 except for 78 DAF. However, in the 2013 vintage, differences in the cultivation modes were only observed at 41 DAF. A total of 11 flavonols were detected. The B-ring 3’4’-substituted flavonols were the major flavonols detected in the grapes (**Table 1**). In the 2012 vintage, the rain-shelter cultivation significantly decreased the level of the B-ring 3’4’-substituted and B-ring 4’-substituted flavonols in most of the grape developmental stages (**Fig 3B**). However, the similar results were not observed in the 2013 vintage grapes. Quercetin-3-*O*-glucoside exhibited the highest level, followed by quercetin-3-*O*-glucuronide, kaempferol-3-*O*-glucoside, and quercetin-3-*O*-galactoside. Compared with the open-field-grown grapes, the rain-shelter-cultivated grapes in the 2012 vintage had lower levels of quercetin-3-*O*-glucoside, kaempferol-3-*O*-glucoside, and quercetin-3-*O*-galactoside at the harvest point, but a higher level of quercetin-3-*O*-glucuronide. Moreover, kaempferol-3-*O*-galactoside and kaempferol-3-*O*-glucuronide showed lower level in the 2012 vintage grapes treated under the rain-shelter application (**Table 1**). The B-ring 3’4’5’-substituted flavonols displayed a downward trend in the total concentration during the grape development process. In the early stages of the 2012 vintage, a significantly higher level of the 3’4’5’-substituted flavonols was observed in the rain-shelter-cultivated grapes (**Fig 3B**). However, both the rain-shelter and the open-field-grown grapes had similar levels of the total 3’4’5’-substituted flavonols at the harvest point, which was due to the dramatic concentration changes of myricetin-3-*O*-glucuronide and myricetin-3-*O*-glucoside in both treatments (**Table 1**). In the 2013 vintage, the rain-shelter and open-field-grown grapes showed the similar concentrations of flavonols. These results indicated that the rain-shelter treatment might down-regulate the accumulation of flavonols in the F3’H and the F3H pathways, but might not affect the ability of the F3’5’H biosynthetic pathway to yield flavonols.

#### Flavan-3-ols

The total flavan-3-ols concentration showed a downward trend during the grape maturation in both of the vintages (**S1C Fig**). The rain-shelter cultivation significantly reduced the level of the total flavan-3-ols in most of the grape developmental stages in 2012. However, such significant treatment differences were not observed in the 2013 vintage grapes. Flavan-3-ols can be grouped into B-ring 3’4’-substituted and 3’4’5’-substituted flavan-3-ols based on flavan-3-ol biosynthesis [14]. B-ring 3’4’-substituted flavan-3-ols are synthesized in the F3’H pathway, whereas the F3’4’H pathway regulates the formation of B-ring 3’4’5’-substituted flavan-3-ols. In this study, the B-ring 3’4’-substituted flavan-3-ols included (+)-catechin (C), (-)-epicatechin (EC), and (-)-epicatechin-3-*O*-gallate (ECG). (-)-Epigallocatechin (EGC) was the only B-ring 3’4’5’-substituted flavan-3-ol detected in these grapes. It should also be noted that the flavan-3-ol polymers were depolymerized with the presence of phloroglucinol, and as a result the flavan-3-ol concentration in this study represented the sum of monomers, terminal, and extension subunits. This could better reflect the effect of the rain-shelter cultivation on the biosynthesis of various flavan-3-ol compounds. In the 2012 vintage, both the B-ring 3’4’-substituted and the 3’4’5’-substituted flavan-3-ols showed a decreasing trend in their total concentration during the grape maturation process (**Fig 3C**). The rain-shelter cultivation consistently kept the B-ring 3’4’-substituted flavan-3-ols at a lower level and the B-ring 3’4’5’-substituted flavan-3-ol at a higher level. In the 2013 vintage, higher levels of both the B-ring 3’4’-substituted and the 3’4’5’-substituted flavan-3-ols were observed in the rain-shelter-cultivated grapes at the early stages. However, such an improvement in the B-ring 3’4’5’-substituted flavan-3-ols disappeared in the rain-shelter-cultivated grapes by the berry maturation point, and a lower level of the B-ring 3’4’-substituted flavan-3-ols was observed in the rain-shelter grapes. These results suggested that rain-shelter cultivation might lower the formation of flavan-3-ols under the F3’4’H-pathway, but promote their biosynthesis via the F3’4’5’H-pathway.

### Effect of rain-shelter cultivation on volatile production

**Fig 4:**
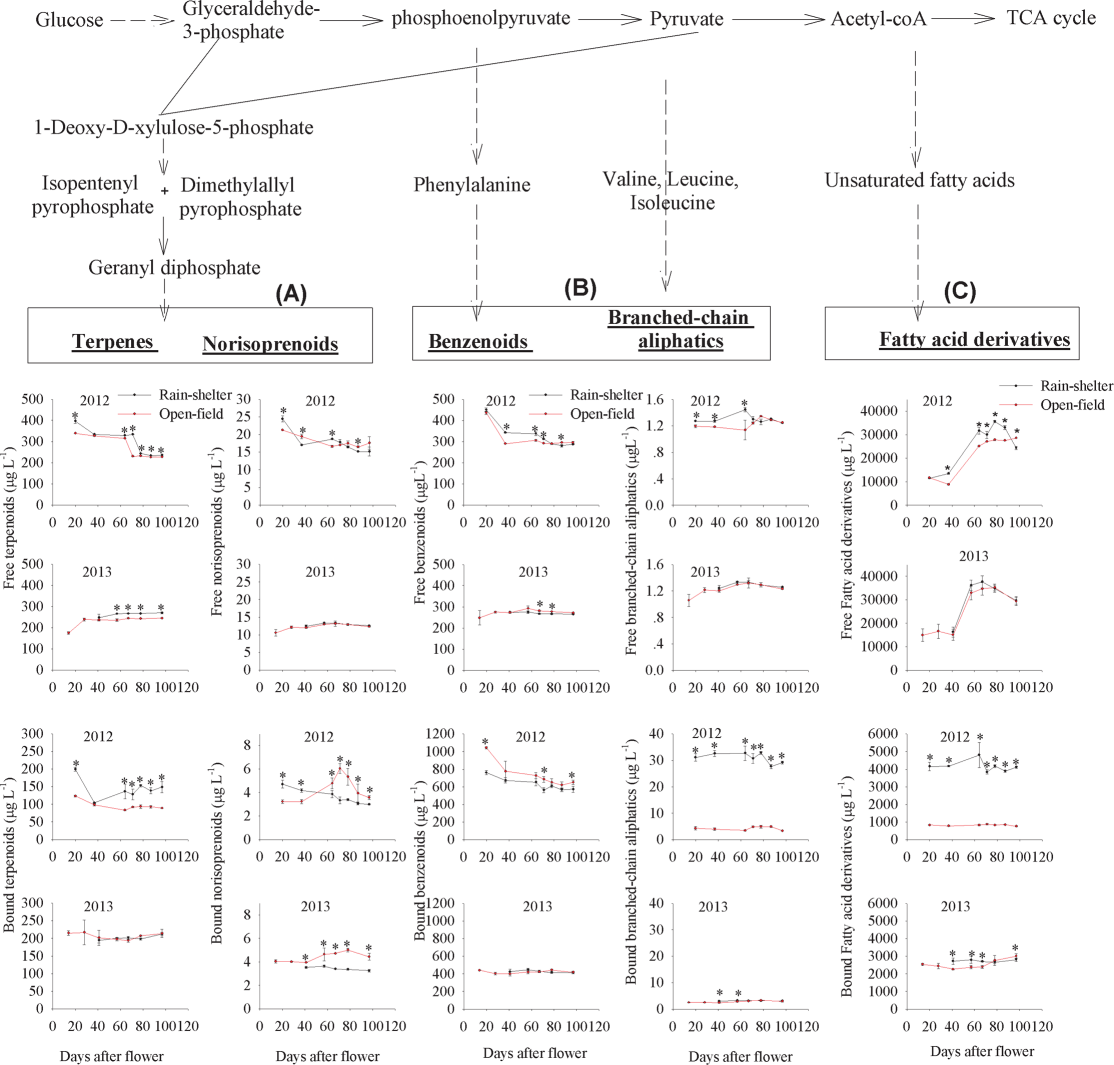
Evolution of free and bound volatile compounds in grapes under the two treatments. (A), (B), and (C) represents the concentrations of free and bound isoprenoid-, amino acid-, and fatty acid-derived volatiles, respectively, under rain-shelter and open-field cultivation in 2012 and 2013. TCA: Tricarboxylic Acid. * represents a significant difference in the concentrations of compounds between the two types of cultivation (*p*<0.05).

Volatiles are abundantly present in grape berries. Free-form volatiles are directly released from berries, whereas bound-form volatiles are released after hydrolysis during processing and storage. To understand the effects of rain-shelter cultivation on the aromatic quality of grape berries used for wine making, we considered both free and glycosidically bound volatiles in the following analyses. According to the synthetic pathways of their precursors, volatiles can be divided into isoprenoid-, amino acid-, and fatty acid-derived volatile compounds (**Fig 4**).

#### Isoprenoid-derived volatile compounds

The total isoprenoid-derived volatiles, including free and glycosidically bound types, gradually decreased during the grape development in the 2012 vintage, but exhibited a slight increase in 2013. Moreover, the rain-shelter cultivation resulted in a remarkable increase in the isoprenoid-derived volatile level in both of the vintages (**S2A Fig**). Terpenoids and norisoprenoids are synthesized from the common C5 isoprene precursors: isopentenyl pyrophosphate (IPP) and its allylic isomer dimethylallyl pyrophosphate (DMAPP). These two precursors are produced in the methyl-erythritol-phosphate (MEP) pathway (**Fig 4A**). In this study, 20 terpenoids and 5 norisoprenoids were detected as their free and bound types in the grapes. The rain-shelter-cultivated grapes showed higher levels of the total free and glycosidically bound terpenoids compared with those under open-field cultivation in the 2012 vintage. Similarly, the rain-shelter cultivation also resulted in the total free terpenoids at a higher level in the 2013 vintage. The total bound-form terpenoids were present in similar levels in the two treatments in 2013. In terms of individual terpenoids at the harvest point, it was observed that most of the glycosidically bound terpenoid compounds differed significantly between the rain-shelter and the open-field-cultivated grapes in the 2012 vintage, but no significant differences were observed in the 2013 vintage grapes (**Table 2**). For instance, the free-form linalool and *D*-limonene, and the bound-form linalool and geraniol displayed higher concentrations in the rain-shelter-cultivated grapes in the 2012 vintage. The rain-shelter cultivation also had a significant impact on the accumulation of most bound norisoprenoids, but no effect was observed on the free norisoprenoid levels (**Fig 4A**). 5-Hepten-2-one, 6-methyl and geranylacetone were the only two bound norisoprenoids detected. Their levels were strikingly reduced in the 2012 rain-shelter-cultivated grapes at the harvest point. However, similar values were observed in both of the cultivated grapes in the 2013 vintage (**Table 2**). These results indicated that rain-shelter cultivation could promote terpenoid metabolism but inhibit norisoprenoid biosynthesis.

**Table 2:** Concentrations of various volatile compounds in the commercially harvested grape berries under rain-shelter and open-field cultivation in the 2012 and 2013 vintages (μg L^-1^).

Numbers	Compounds	Free compounds				Bound compounds			
		2012		2013		2012		2013	
		Open-field	Rain-shelter	Open-field	Rain-shelter	Open-field	Rain-shelter	Open-field	Rain-shelter
**Fatty acid-derivated volatiles**									
**——Alocohol compounds**									
a1	1-pentanol	7.32±1.61	5.74±0.56	2.29±0.30	3.10±0.82	nd	nd	nd	nd
a2	2-heptanol	**tr**	**0.17±0.01**	tr	tr	**5.61±0.16**	**22.90±0.63**	3.98±0.93	4.36±0.43
a3	1-hexanol	42.62±1.22	44.62±0.75	154.75±22.85	169.55±70.33	**44.31±1.07**	**305.90±9.40**	327.31±53.39	329.68±14.71
a4	(E)-3-hexen-1-ol	1.54±0.26	1.85±0.154	0.60±0.08	0.74±0.15	**0.58±0.053**	**5.50±0.15**	0.57±0.21	0.59±0.06
a5	(Z)-3-hexen-1-ol	**3.93±0.65**	**15.20±0.34**	1.02±0.18	1.23±0.18	**11.25±0.38**	**53.78±0.518**	3.95±0.66	4.07±0.50
a6	3-octanol	1.27±0.00	1.27±0.01	1.26±0.00	1.26±0.00	tr	tr	1.95±0.69	2.27±0.16
a7	(E)-2-hexen-1-ol	**2.97±0.22**	**7.70±1.74**	tr	tr	**6.66±0.37**	**22.28±9.02**	1.321±0.15	1.40±0.06
a8	(Z)-2-hexen-1-ol	0.49±0.07	0.38±0.10	tr	tr	**0.18±0.02**	**0.79±0.02**	0.18±0.01	0.18±0.01
a9	1-octen-3-ol	**1.11±0.00**	**0.93±0.02**	0.63±0.14	0.69±0.22	**0.96±0.01**	**4.89±0.11**	0.82±0.13	0.86±0.03
a10	1-heptanol	**3.85±0.05**	**3.76±0.02**	3.53±0.03	3.55±0.02	**3.80±0.13**	**17.06±0.88**	1.74±0.50	1.97±0.19
a11	1-octanol	6.11±0.05	6.13±0.03	5.94±0.02	5.97±0.02	**4.79±0.04**	**20.39±0.68**	4.34±0.35	4.42±0.11
a12	(E)-2-octen-1-ol	4.62±0.58	4.50±0.46	**0.01±0.01**	**0.35±0.25**	tr	tr	2.44±1.21	2.95±0.76
a13	1-dodecanol	**1.98±0.05**	**1.78±0.01**	1.68±0.01	1.67±0.01	tr	tr	2.32±0.03	2.33±0.03
a14	(Z)-3-nonen-1-ol	tr	tr	tr	tr	**15.25±2.75**	**46.57±3.78**	4.19±1.20	5.04±0.83
a15	1-decanol	10±0.10	10.05±0.10	tr	tr	**0.48±0.15**	**2.34±0.61**	0.30±0.05	0.26±0.02
a16	2-nonanol	tr	tr	tr	tr	tr	tr	0.29±0.01	0.29±0.00
a17	2-octanol	tr	tr	tr	tr	**0.40±0.01**	**2.30±0.02**	0.34±0.03	0.36±0.01
a18	1-butanol	tr	tr	tr	tr	**582.48±7.74**	**690.51±36.07**	579.03±26.65	586.64±8.73
**——Aldehyde compounds**									
a19	hexanal	**9356.40±100.54**	**7872.23±263.65**	9365.79±543.35	9181.83±795.55	nd	nd	nd	nd
a20	(E)-2-hexenal	**18314.60±11.19**	**15473.53±223.16**	14495.44±958.25	15051.73±964.25	nd	nd	nd	nd
a21	octanal	**5.31±0.02**	**5.05±0.05**	4.95±0.07	4.35±0.14	**5.94±0.47**	**tr**	4.74±0.26	4.55±0.09
a22	(E)-2-heptenal	**10.42±0.84**	**8.27±0.15**	**2.47±0.28**	**3.06±0.51**	nd	nd	nd	nd
a23	decanal	1.06±0.04	1.02±0.03	0.74±0.03	0.64±0.04	nd	nd	nd	nd
a24	(E)-2-octenal	7.80±0.24	10.75±3.12	tr	tr	nd	nd	nd	nd
a25	nononal	**11.21±0.05**	**10.89±0.04**	10.87±0.11	10.86±0.07	**0.83±0.11**	**3.24±0.06**	0.65±0.07	0.58±0.03
**——Acid compound**									
a26	hexanoic acid	**92.29±1.3**	**78.65±0.35**	75.78±0.31	76.07±1.72	nd	nd	nd	nd
**——Ester compounds**									
a27	ethyl hexanoate	2.79±0.05	3.05±0.23	2.70±0.01	2.72±0.01	**0.94±0.84**	**5.34±0.47**	tr	tr
a28	hexyl acetate	5.46±0.01	5.45±0.02	5.42±0.01	5.42±0.02	**tr**	**6.56±0.26**	tr	tr
a29	(Z)-3-hexen-1-ol, acetate	5.69±0.04	5.72±0.02	5.45±0.01	5.47±0.03	tr	tr	1.81±0.07	1.82±0.02
a30	ethyl octanoate	**4.89±0.42**	**7.87±1.11**	**4.29±0.00**	**4.32±0.02**	2.88±3.07	4.74±2.96	tr	tr
a31	ethyl decanoate	2.99±0.03	3.04±0.08	**0.48±1.18**	**tr**	tr	tr	tr	tr
a32	butyl butyrate	**2.81±0.01**	**2.79±0.01**	2.72±0.00	2.72±0.01	tr	tr	3.09±0.17	3.13±0.13
**Amino acid-derivated volatiles**									
**——Benzenoids**									
b1	styrene	**9.91±0.02**	**10.03±0.04**	**8.48±0.06**	**8.70±0.07**	4.82±0.17	4.78±0.32	3.89±0.08	3.95±0.03
b2	furfural	1.37±0.01	1.29±0.07	1.14±0.02	1.15±0.02	5.12±0.30	5.03±0.03	tr	tr
b3	benzaldehyde	**41.87±2.56**	**17.31±0.25**	**39.81±5.68**	**tr**	6.79±3.67	4.90±0.14	18.31±0.37	18.23±0.99
b4	5-methyl furfural	**8.62±0.09**	**8.44±0.02**	8.46±0.02	8.48±0.02	tr	tr	tr	tr
b5	benzylethylaldehy	**24.39±0.30**	**18.45±0.23**	35.42±3.30	36.87±4.14	4.25±0.68	4.05±0.34	5.26±0.17	4.93±1.95
b6	ethyl benzoate	**1.98±0.01**	**1.93±0.00**	1.88±0.01	1.88±0.01	tr	tr	tr	tr
b7	naphthalene	**8.76±0.11**	**6.21±0.23**	**5.65±0.32**	**6.98±0.50**	**15.52±1.49**	**10.02±0.46**	7.70±1.27	7.76±0.93
b8	TDN	tr	tr	tr	tr	nd	nd	nd	nd
b9	ethyl salicylate	tr	tr	tr	tr	1.23±0.01	1.23±0.00	tr	tr
b10	methyl salicylate	**5.96±0.01**	**6.3±0.04**	5.61±0.01	5.62±0.01	tr	tr	0.75±0.02	0.78±0.04
b11	benzaldehyde, 3,4-dimethyl	**25.20±0.05**	**24.87±0.01**	25.04±0.10	25.11±0.09	**5.65±1.88**	**9.21±1.67**	3.19±0.01	3.16±0
b12	benzyl alcohol	16.55±0.43	16.03±0.08	**14.94±0.10**	**15.29±0.22**	**261.64±12.82**	**239.03±16.75**	**168.09±4.92**	**119.07±9.25**
b13	aphthalene,1-methyl-	10.72±0.12	10.65±0.01	10.60±0.01	10.63±0.01	**3.41±0.18**	**2.05±0.09**	2.57±0.51	2.71±0.21
b14	2-phenylethanol	125.45±0.09	124.67±1.27	123.60±0.04	123.69±0.6	332.92±11.22	330.33±0.92	267.62±8.20	264.40±3.27
b15	4-methylphenol	10.63±0.11	10.58±0.01	10.53±0.01	10.53±0.01	**6.80±0.700**	**4.94±0.34**	tr	tr
b16	2,6-diterbutyl-4-methyl phenol	tr	tr	tr	tr	2.66±0.03	2.72±0.03	0.52±0.13	0.57±0.08
b17	4-ethyl phenol	tr	tr	8.77±4.29	10.52±0.01	**0.91±0.05**	**3.75±1.05**	0.62±0.05	0.64±0.02
b18	phenol	**4.45±0.14**	**3.64±0.01**	**2.85±0.03**	**2.96±0.07**	**2.03±0.31**	**1.09±0.04**	2.64±0.01	2.64±0.00
**——Branched-chain aliphatics**									
c1	2-ethyl-1-hexanol	1.25±0.01	1.25±0.01	1.23±0.02	1.26±0.01	**2.80±0.08**	**17.98±0.32**	2.59±0.35	2.69±0.04
c2	4-methyl-1-pentanol	tr	tr	tr	tr	**0.52±0.095**	**11.36±0.17**	0.36±0.10	0.40±0.02
**Isoprenoid-derivated volatiles**									
**——Terpenoids**									
d1	β-myrcene	tr	tr	tr	tr	17.88±0.07	17.78±0.05	18.03±0.29	17.95±0.09
d2	D-limonene	**8.36±0.09**	**11.61±0.01**	8.27±0.14	8.30±0.07	2.90±0.27	2.66±0.28	3.27±0.34	3.21±0.03
d3	p-cymene	tr	tr	tr	tr	5.57±0.03	5.59±0.01	5.54±0.02	5.54±0.01
d4	TCH	tr	tr	4.18±0.00	3.48±1.71	nd	nd	nd	nd
d5	γ-terpinene	tr	tr	tr	tr	2.05±0.42	1.92±1.51	2.79±0.75	2.65±0.23
d6	terpinolene	**tr**	**5.28±0.01**	5.29±0.03	5.29±0.02	**4.25±0.03**	**tr**	4.29±0.03	4.29±0.01
d7	(Z)-furan linalool oxide	**1.48±0.04**	**1.20±0.01**	**0.42±0.09**	**19.50±0.17**	**1.63±0.16**	**2.91±0.11**	1.52±0.17	1.57±0.37
d8	(E)-furan linalool oxide	tr	tr	0.18±0.04	0.16±0.01	**0.70±0.07**	**1.25±0.05**	0.65±0.07	0.61±0.052
d9	nerol oxide	tr	tr	1.76±0.01	1.77±0.01	**0.27±0.01**	**0.15±0.00**	0.24±0.02	0.25±0.02
d10	linalool	**27.87±0.09**	**28.70±0.05**	27.85±0.42	27.86±0.25	**7.09±0.11**	**12.77±0.28**	8.37±2.12	7.79±0.30
d11	4-terpinenol	6.24±0.01	6.25±0.01	6.21±0.00	6.21±0.01	**0.78±0.01**	**0.86±0.01**	0.74±0.03	0.75±0.01
d12	hotrienol	26.33±0.11	26.52±0.32	26.27±0.01	26.28±0.01	9.37±0.01	9.37±0.01	tr	tr
d13	β-cyclocitral	4.2±0.00	4.2±0.00	4.18±0.00	4.18±0.01	tr	tr	tr	tr
d14	menthol	6.37±0.02	6.35±0.02	6.36±0.17	6.27±0.04	**1.31±0.03**	**1.09±0.03**	0.97±0.11	1.02±0.08
d15	α-terpineol	6.36±0.12	6.46±0.01	**6.28±0.04**	**tr**	**tr**	**1.96±0.05**	0.44±0.05	0.45±0.02
d16	geranial	0.99±0.08	0.94±0.04	tr	tr	**1.41±0.02**	**1.94±0.19**	1.28±0.05	1.29±0.02
d17	benzenemethanol	tr	tr	tr	tr	0.70±0.08	0.66±0.00	tr	tr
d18	neral	tr	tr	tr	tr	**3.48±0.05**	**3.36±0.01**	3.38±0.02	3.39±0.01
d19	nerol	3.72±0.10	3.86±0.02	tr	tr	**10.09±0.17**	**9.67±0.12**	7.89±0.59	7.83±0.22
d20	geraniol	tr	tr	tr	tr	**17.76±0.32**	**24.51±0.45**	9.62±0.88	9.41±0.38
**——Norisoprenoids**									
f1	5-hepten-2-one,6-methyl	4.40±0.14	4.23±0.02	4.19±0.01	4.19±0.01	**1.55±0.13**	**1.16±0.01**	1.42±0.21	1.41±0.02
f2	β-damascenone	**5.26±0.34**	**4.73±0.01**	4.49±0.17	4.67±0.40	nd	nd	nd	nd
f3	α-ionone	tr	tr	3.33±0.00	3.33±0.01	nd	nd	nd	nd
f4	geranylacetone	3.66±1.50	1.92±0.89	0.88±0.04	0.87±0.04	**2.04±0.06**	**1.80±0.01**	1.94±0.14	1.84±0.09
f5	β-ionone	4.27±0.01	4.25±0.01	4.18±0.00	4.19±0.01	nd	nd	nd	nd

tr: trace; nd: not detected

Bold & underline represents that the compund concentration has statistically-significant difference between the rain-shelter and open-field grapes in the same vintage (p<0.05)

#### Amino acid-derived volatile compounds

Amino acid-derived volatiles include benzenoids, branched-chain aliphatics, and methoxypyrazines. Benzenoids are synthesized from phenylalanine, whereas branched-chain aliphatics and methoxypyrazines are the products of three branched-chain amino acids: valine, isoleucine and leucine [23]. The total concentration of the amino acid-derived volatiles gradually decreased during the grape development in both vintages, and no significant differences in concentration were observed between the two cultivated grapes (**S2B Fig**). In this study, 18 benzenoids and 2 branched-chain aliphatics were detected in the grapes. It should also be noted that 2 methoxypyrazines, i.e., 3-isobutyl-2-methoxypyrazine (IBMP), and 3-isopropyl-2-methoxypyrazine (IPMP), were only present in the rain-shelter -cultivated grapes in the early development stages (0–40 DAF). Although the rain-shelter cultivation had different impacts on the free benzenoids in the unripe grapes of the two vintages, there was almost no difference in the total free benzenoid concentration in the commercially ripe grapes cultivated under these two modes. The rain-shelter cultivation resulted in a lower level of glycosidically bound benzenoids in the 2012 vintage grapes but did not cause an obvious change in these benzenoids in the 2013 vintage grapes (**Fig 4B**). Free-form 2-phenylethanol, benzaldehyde, 3,4-dimethyl benzaldehyde, and benzylethylaldehyde were the major free benzenoids detected in the grapes. Except for 2-phenylethanol, these compounds were present at a lower level in the 2012 rain-shelter grapes. However, except for benzaldehyde, they were present at a similar level in the rain-shelter and open-field grapes in 2013. Additionally, it was observed that the bound-form benzyl alcohol had a much higher level in the 2012 vintage grapes than in the 2013 vintage grapes. This explained the significant difference in the total glycosidically bound benzenoid level between these two vintages (**Table 2**). These results suggested that rain-shelter cultivation might down-regulate the biosynthesis of benzenoids. Regarding the branched-chain aliphatics, their bound form indicated a much higher level in the rain-shelter grapes at the harvest point of the 2012 vintage, but similar levels were observed for the two cultivation modes in the 2013 vintage (**Table 2**). The free branched-chain aliphatics had a low level in both of the two vintage grapes and the rain-shelter cultivation did not significantly change their accumulation (**Fig 4B**).

#### Fatty acid-derived volatile compounds

The total concentration of the fatty acid-derived volatiles initially increased and then decreased with the grape development. The highest concentration of the total fatty acid-derived volatiles was observed at the end of veraison (87 DAF in 2012, 78 DAF in 2013). Compared with the open-field grapes, the rain-shelter-cultivated grapes exhibited a higher level of the fatty acid-derived volatiles during 40 to 87 DAF in the 2012 vintage, but had a lower level at the harvest point. The 2013 vintage grapes under both cultivation modes exhibited a similar level of the fatty acid-derived volatiles (**S2C Fig**). Among the three groups (isoprenoid-, amino acid-, and fatty acid-derived compounds), the fatty acid-derived volatiles accounted for the highest proportional concentration (**Fig 4C)**. It was observed that the changes in the total fatty acid-derived volatiles during the grape maturation resulted mainly from the changes in the free-form compounds. In the 2012 vintage, the unripe grapes under the rain-shelter cultivation had a higher level of free fatty acid-derived volatiles, but their level significantly decreased when the grapes approached maturation. As a result, its level was lower than that in the open-field grapes at the harvest point. In 2013, the rain-shelter treatment did not result in the significant differences in the free fatty acid-derived volatile levels. The concentrations of the bound fatty acid-derived volatiles varied dramatically in both vintages. The rain-shelter cultivation greatly increased the accumulation of the glycosidically bound fatty acid-derived volatiles in the 2012 vintage compared with the open-field cultivation. The enhancement effect in the 2013 vintage was considerably less than that in the 2012 vintage (**Fig 4C**).

The fatty-acid derived volatiles detected in this study included 18 straight-chain aliphatic alcohols, 7 straight-chain aldehydes, 1 straight-chain acid, and 6 straight-chain esters (**Table 2**). The straight-chain aldehydes had the highest concentration, which accounted for more than 80% of the total fatty acid-derived volatiles. These aldehydes accumulated differently during the development of the two types of cultivated grapes, causing the difference in the total concentration of the fatty acid-derived volatiles. Among the straight-chain aliphatic aldehydes, hexanal and (*E*)-2-hexenal exhibited the highest levels and their concentrations in the rain-shelter grapes were significantly lower than those in the open-field grapes at the harvest point of the 2012 vintage. However, no significant differences in their concentration occurred between the two treatments in the 2013 vintage. For the straight-chain aliphatic alcohols, the rain-shelter cultivation significantly increased the levels of the free and the glycosidically bound alcohols as well as the glycosidically bound aldehydes and esters, compared with the open-field cultivation in the 2012 vintage. In addition, the rain-shelter cultivation also reduced the levels of the free-form aldehydes, esters, and acids. However, the straight-chain aliphatic volatiles did not show any significant difference between the two cultivation approaches at the harvest point of the 2013 vintage (**Table 2**).

The results above indicated that rain-shelter cultivation up-regulated the isoprenoid pathway, especially the terpene formation in both vintages. Additionally, this cultivation approach could also promote the biosynthesis of branched-chain amino acid-derived volatiles, which resulted in the higher level in the 2012 vintage grapes. The concentrations of the other volatile metabolites from fatty acids and phenylalanine were not altered by the rain-shelter cultivation at the harvest point.

### Principal component analysis

**Fig 5:**
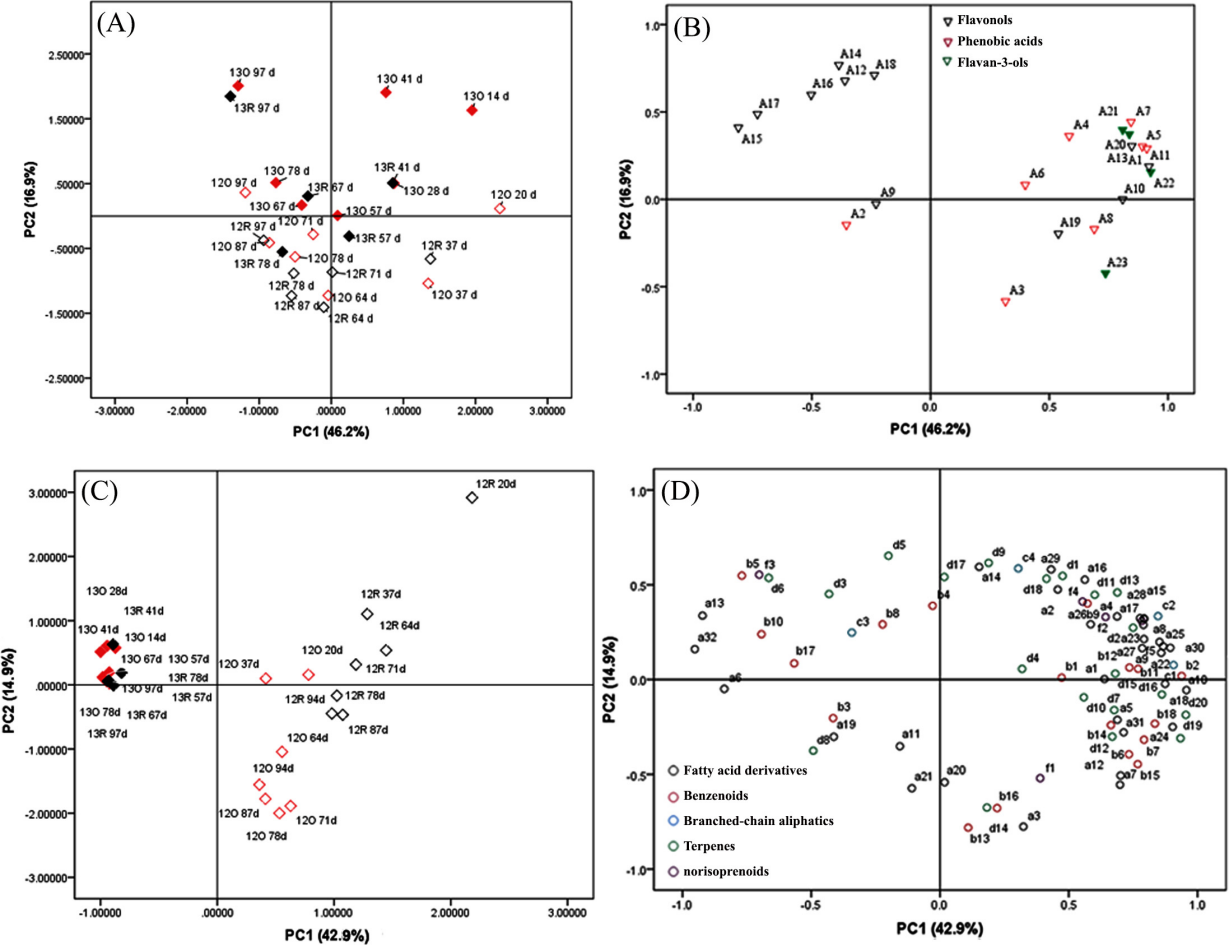
Principal component scatter plot and corresponding loading plot of polyphenols and volatiles. ‘R’ and ‘O’ represent rain-shelter and open-field cultivation, respectively.

To provide an overview of the effects of the vintage and the cultivation treatments on the polyphenol and volatile profiles and to further identify the discriminant components, principal component analysis (PCA) was applied using all of the detected phenolic and volatile compounds as variables, taking into account all sampling dates for each treatment. The PCA score scatter plots of all of the grape samples are shown in **Fig 5A** (based on phenolic variables) and **Fig 5C** (based on volatile variables), and the corresponding loading plots establishing the relative importance of the variables are listed in **Fig 5B** (phenolic variables) and **Fig 5D** (volatile variables). The combination of the scatter plots and loading plots reflects the corresponding relationship between the grape samples and their phenolic or volatile profile. Based on all of the detected phenolic compounds, the first two principal components (PCs) explained approximately 63.1% of the total variance. The first component (PC1) accounted for approximately 46.2% and the samples were distributed from the positive to the negative axis of PC1, corresponding to the grape developmental stages. PC2 accounted for 16.9% of the total variance. Except for two samples (13R 57d and 13R 78d, which were collected at 57 and 78 DAF, respectively, from the 2013 vintage under the rain shelter), all of the 2013 vintage samples were distributed in the first and second quadrants, corresponding to the positive axis of PC2. The 2012 vintage samples were mostly concentrated in the third and fourth quadrants, corresponding to the negative axis of PC2 (**Fig 5A**). In combination with the corresponding loading plot, it was observed that the 2013 vintage grapes, compared with the 2012 vintage grapes, were characterized by higher levels of phenolic acid compounds (e.g., caffeic acid (A5) and *p*-coumaric acid (A7)) and flavan-3-ols (e.g., (+)-catechin (A20) and (-)-epicatechin (21)) at the early developmental stage, and higher level of flavonols (e.g., quercetin-3-*O*-glucoside (A14) and kaempferol-3-*O*-galactoside (15)) at the later stage (**Fig 5B**). It should be noted here that the grape samples under these two cultivation treatments could not be well differentiated by PC1 and PC2 based on their phenolic compounds. To determine which phenolic compounds were essentially affected by the cultivation treatments, we performed two-way variance analysis regarding the vintage and cultivation treatment, using all the data from the various developmental stages (**Table 3**). The results revealed that five compounds exhibited a significant difference in their concentration between the two cultivation treatments, i.e., dihydroquercetin-3-*O*-glucoside (A9), myricetin-3-*O*-glucoside (A11), quercetin-3-*O*-glucuronide (A13), quercetin-3-*O*-glucoside (A14), and (+)-catechin (A20) (**Table 3**). The rain-shelter cultivation caused a decrease in these compounds, except for quercetin-3-*O*-glucuronide. The variance analysis also demonstrated that various phenolic acids were not influenced by the rain-shelter cultivation. In addition, 12 compounds showed a significant difference between the vintages. It is beyond all doubt that the vintage, relative to the rain cultivation, affected the grape phenolic profile more significantly. Four compounds were jointly affected by both vintage and cultivation treatment.

**Table 3:** The results (F-value) of two-way analysis of variance on the effects of vintage and cultivation on various polyphenols in grape berries.

Numbers	Phenolic compounds	Vintage	Cultivation	Vintage*Cultivation
A1	Gallic acid	0.162	1.198	0.167
A2	Protocatechuic acid	9.236**	0.039	0.587
A3	*p*-Hydroxybenzoic acid	52.021**	0.101	0.234
A4	Chlorogenic acid	1.368	0.623	1.215
A5	Caffeic aci	2.758	0.410	1.038
A6	Syringic acid	1.182	0.747	0.558
A7	*p*-Coumaric acid	10.848**	0.803	0.231
A8	Ferulic acid	15.827**	3.584	3.512
A9	Dihydroquercetin-3-*O*-glucoside	3.151	7.746**	4.215*
A10	Myricetin-3-*O*-glucuronide	9.991**	0.888	9.267**
A11	Myricetin-3-*O*-glucoside	0.660	4.264*	0.072
A12	Quercetin-3-*O*-galactoside	3.912	3.918	3.301
A13	Quercetin-3-*O*-glucuronide	0.004	6.327*	2.158
A14	Quercetin-3-*O*-glucoside	5.341*	6.410*	0.495
A15	Kaempferol-3-*O*-galactoside	1.405	1.020	0.592
A16	Kaempferol-3-*O*-glucuronide	8.766**	3.546	0.127
A17	Kaempferol-3-*O*-glucoside	1.110	1.308	0.718
A18	Isorhamnetin-3-*O*-glucoside	8.524**	1.425	5.578*
A19	Quercetin	31.407**	0.305	0.222
A20	Catechin	12.787**	7.322**	0.363
A21	Epicatechin	23.376**	2.718	0.505
A22	Epigallocatechin	0.310	0.012	4.794*
A23	Epicatechin-3-O-gallate	42.508**	0.897	0.552

** a significance level of p<0.01

* a significance level of p<0.05

The volatile variables used in the PCA consisted of the total concentration of their respective free and glycosidically bound types. Based on all of the volatile variables detected in this study, the first two components explained 57.8% of the total variance, with the first component accounting for 42.9%. The 2012 vintage samples were clearly separated from the 2013 samples in accordance with PC1 (**Fig 5C**). From the loading plot (**Fig 5D**), it was observed that the majority of the volatile compounds were loaded onto the positive axis of PC1 (the first and fourth quadrants), which indicated that these compounds generally had higher levels in the 2012 vintage grapes compared with the 2013 vintage grapes. The compounds loaded onto the negative axis of PC1 were composed of a few straight-chain aliphatic alcohols and esters. These compounds showed higher levels in the 2013 vintage grapes. Moreover, the samples from the two cultivation treatments of the 2012 vintage were also well differentiated, and the rain-shelter samples showed higher PC1 scores. The discrimination between the two treatments was mainly related to straight-chain aliphatic aldehydes and terpenes. However, the 2013 vintage samples were not clearly differentiated according to their cultivation modes, particularly at the late developmental stages (67–97 DAF) (**Fig 5C**). These indicated that the effect of the rain-shelter cultivation on the grape volatile profile largely depended on the vintage. The two-way analysis of variance revealed that 68 volatile compounds exhibited a significant difference between the vintages, as 35 compounds did between the cultivation treatments (**Table 4**). According to the F-values, it was inferred that eight compounds were more strongly affected by the cultivation treatment in comparison with the vintage. These volatile compounds included two fatty acid-derivatives (1-hexanol and octanal), three benzenoids (benzaldehyde, 3,4-dimethyl benzaldehyde, and *trans*-furan-1-methyl aphthalene), and three terpenoids ((*E*)-furan linalool oxide, nerol oxide, and neral) (**Table 4**). From the perspective of the whole grape developmental period, the rain-shelter cultivation resulted in a reduction in the production of these compounds except for 1-hexanol and (*E*)-furan linalool oxide.

**Table 4:** The results (F-value) of two-way analysis of variance on the effects of vintage and cultivation on volatile compounds in grape berries.

Numbers	Volatile compounds	Vintage	Cultivation	Vintage*Cultivation
a1	1-pentanol	63.43**	0.070	.000
a2	2-heptanol	50.28**	13.04**	2.9
a3	1-hexanol	0.590	11.49**	10.42**
a4	(E)-3-hexen-1-ol	68.64**	52.73**	86.79**
a5	(Z)-3-hexen-1-ol	61.35**	4.12*	6.65*
a6	3-octanol	187.85*	0.23	0.21
a7	(E)-2-hexen-1-ol	160.92**	34.65**	33.12**
a8	(Z)-2-hexen-1-ol	86.24**	26.77**	26.71**
a9	1-octen-3-ol	553.01**	414.47**	391.13**
a10	1-heptanol	49.95**	17.68**	13.58**
a11	1-octanol	64.22**	33.65**	17.75**
a12	(E)-2-octen-1-ol	80.45**	6.22*	0.05
a13	1-dodecanol	368.89**	0.33	2.24
a14	(Z)-3-nonen-1-ol	8.17**	4.94*	0.03
a15	1-decanol	138.01**	41.67**	42.79**
a16	2-nonanol	13.49**	2.61	2.56
a17	2-octanol	46.59**	14.25**	9.1**
a18	1-butanol	117.82**	0.55	0.45
a19	hexanal	29.11**	1.33	0.28
a20	(E)-2-hexenal	1.62	0.89	0.11
a21	octanal	41.69**	703.1**	20.62**
a22	(E)-2-heptenal	196.09**	2.85	0.61
a23	decanal	6.42*	0.15	0.08
a24	(E)-2-octenal	136.68**	0.92	1.51
a25	nononal	308**	134.31**	130.85**
a26	hexanoic acid	22.29**	0.11	0.15
a27	ethyl hexanoate	432.96**	303.43**	302.87**
a28	hexyl acetate	1182.63**	926.84**	918.43**
a29	(Z)-3-hexen-1-ol, acetate	4.99*	2.38	1.77
a30	ethyl octanoate	212.63**	75.79**	75.51**
a31	ethyl decanoate	76.67**	6.83*	13.51**
a32	butyl butyrate	1193.62**	0.02	3.05
b1	styrene	22**	1.99	0.07
b2	furfural	269.26**	0.73	0.67
b3	benzaldehyde	23.51**	160.81**	36.84**
b4	5-methyl furfural	0	0.61	0.74
b5	benzylethylaldehy	1.71	1.43	1.17
b6	ethyl benzoate	86.02**	0.02	0.11
b7	naphthalene	151.63**	0.55	1.12
b8	TDN	0.12	0.17	4.82*
b9	ethyl salicylate	13.36**	0.34	0.34
b10	methyl salicylate	22.91**	1.8	2.08
b11	benzaldehyde, 3,4-dimethyl	5.37*	118.3**	70.82**
b12	benzyl alcohol	50.38**	0.05	3.39
b13	aphthalene,1-methyl-	5.34*	25.46**	39.4**
b14	2-phenylethanol	77.92**	5.25*	7*
b15	4-methylphenol	191.36**	10.72**	10.73**
b16	2,6-diterbutyl-4-methyl phenol	12.93**	9.64**	9.49**
b17	4-ethyl phenol	226.44**	4.86*	2.42
b18	phenol	280.73**	3.58	7.07*
c1	2-ethyl-1-hexanol	219.76**	140.82**	118.17**
c2	4-methyl-1-pentanol	45.54**	22.24**	20.15**
c3	IBMP	5.99*	5.99*	8.41**
c4	IPMP	0.63	10.29**	0.63
d1	β-myrcene	17.44**	2.79	3.48
d2	D-limonene	63.2**	47.6**	46.48**
d3	p-cymene	7.11*	3.03	4.72*
d4	TCH	5.08*	0.88	1.12
d5	γ-terpinene	2.77	1.73	0.56
d6	terpinolene	74.41**	0.74	0.74
d7	cis-furan linalool oxide	35.63**	4.37*	0.07
d8	trans-furan linalool oxide	27.82**	77.29**	49.75**
d9	nerol oxide	1.6	10.44**	10.25**
d10	linalool	21.22**	3.64	5.03*
d11	4-terpinenol	12.55**	2.8	2.58
d12	hotrienol	393.01**	0.87	5.09*
d13	β-cyclocitral	22.67**	1.18	0.8
d14	menthol	16.55**	14.5**	12.71**
d15	α-terpineol	19.89**	3.72	16.41**
d16	geranial	200.01**	0.11	0.06
d17	benzenemethanol	40.01**	14.94**	14.94**
d18	neral	1.95	16.05**	22.6**
d19	nerol	7.06*	0.07	0.08
d20	geraniol	1.98	0.01	0
f1	5-hepten-2-one,6-methyl	10.93**	0.92	1.78
f2	β-damascenone	13.45**	0.15	0.34
f3	α-ionone	104.51**	0.86	0.86
f4	geranylacetone	47.22**	0.99	1.37
f5	β-ionone	39.16**	1.06	0.57

** a significance level of p<0.01

* a significance level of p<0.05

### Climate characteristics of the two vintages

The meteorological data at the experimental site were recorded for the 2012 and the 2013 vintages. There existed some differences in the total rainfall (524.2 mm and 465.6 mm) and the total sunshine duration (579.9 h and 687.0 h) throughout the whole period of the grape development in 2012 and 2013. However, slight differences also existed in the daily average temperature (24.5°C and 24.3°C) and the daily average temperature difference (10.6°C and 9.8°C). To understand the climate characteristics during the various developmental phases, we assessed the meteorological data for the first rapid growth phase (from bloom to 60 DAF; Stage I), veraison (lag) phase (approximately 61–80 DAF; Stage II), and berry maturation phase (81–97 DAF; Stage III). Compared with the same period in 2013, the veraison phase of the 2012 vintage had nearly one-fold higher rainfall, but its berry maturation phase received nearly two-fold less rainfall. Correspondingly, there was a longer sunshine duration and a higher average daily temperature in the berry maturation phase of the 2012 vintage, whereas the sunshine duration in the first rapid phase of 2012 was shorter than that in 2013 (**Table 5**).

**Table 5:** Main meteorological parameters of Miyun, Beijing, in different grape development periods in 2012 and 2013.

Grape development periods	Rainfall /mm		Sunshine hours /h		Average temperature /°C		Average daily temperature difference /°C	
	2012	2013	2012	2013	2012	2013	2012	2013
Stage I	343.1	330.2	357.7	450	24.4	24.8	10.5	9.6
Stage II	164.2	86.1	88.7	117.4	25.7	26.2	7.9	9.9
Stage III	16.9	49.3	133.5	119.6	23.7	21.2	11.2	10.7

## Discussion

Rain-shelter cultivation aims to reduce disease occurrence and improve berry quality. Our previous studies reported that simple rain-shelter application prolonged the lifetime of functional leaves and enhanced the accumulation of sugar in Cabernet Sauvignon grapes cultivated on the East Coast of China [7]. Detonni and his colleagues also reported that the soluble solids content was improved in Cabernet Sauvignon grapes under a rain shelter in southern Brazil compared with those grown in an open field [15]. However, a similar rain-shelter treatment in this study did not significantly increase the accumulation of soluble solids in Chardonnay grapes grown in the Huaizhuo basin region of northern China. These might be attributed to different grape varieties and/or local climate. In the Huaizhuo basin region, we also found that rain-shelter cultivation significantly increased the soluble solids content of Cabernet Sauvignon grapes (unpublished data).

Rain-shelter cultivation has been confirmed to alter the microclimate around the canopy and grape cluster, consequently increasing the air temperature, leaf stomatal conductance and CO_2_ level, but reducing the photosynthetically active radiation, wind speed, and moisture from transpiration around grape berries [2, 4]. These effects are mainly dependent on the rain-shelter structure. The simple rain shelter applied in the present study only reduced the solar radiation, photosynthetically active radiation and ultraviolet radiation reaching the grape clusters at midday, but had no detectable effect on the other meteorological factors. Previous studies reported that visible light primarily promoted the biosynthesis of proanthocyanidins, whereas UV light particularly up-regulated the biosynthesis of flavonols [24]. Moreover, the effects of light on the accumulation of proanthocyanidins and flavonols in grapes were much greater at the early stages of grape development [18, 25]. Our present study also revealed that rain-shelter cultivation resulted in a decrease in the sum of flavonols and flavan-3-ols at the early development stage of Chardonnay grapes, which might be a consequence of the reduction in solar radiation inside the rain shelter. However, in terms of their biosynthetic pathways, it was observed that the 3’4’5’- substituted flavanols and the flavan-3-ols from the F3’5’H pathway were enhanced in the 2012 rain-shelter grapes, and the 3’4’5’- substituted flavan-3-ols were also improved in the 2013 rain-shelter grapes at the early developmental stage. This result indicated that changes in light conditions around the grapes caused by rain-shelter application could alter the metabolic carbon flow into two branch pathways during flavonoid biosynthesis. This modification of carbon flow eventually decreased the 3’4’-substituted flavonoid contribution to the phenolic profiles in the rain-shelter-cultivated grapes. There are very few reports available on the effect of solar radiation on metabolic carbon flow in the flavonoid pathway. A previous study showed that shading treatment applied to grapes at pre-veraison significantly lowered the transcript abundance of *VvF3′5′H* and *VvF3′H* and altered the distribution of 3’4’-substituted flavan-3-ols in ripe grapes [14]. This suggested that the shading treatment had a greater effect on the down-regulation of the F3’H pathway than on the F3’5’H pathway during grape development. Additionally, another study demonstrated that the transcript level of *VvF3′5′H* was significantly decreased in grapes after 3 days of shading treatment, which might result from the improvement of visible light on the transcript abundance of this gene [24]. Furthermore, *VvCYTB5* has been considered as a candidate for the modulation of *VvF3’H* and *VvF3’5’H* expressions in *Vitis vinifera* L. cv Shiraz grapes, and it was found that grapes subjected to a lack of light caused by light-proof boxes expressed this gene at a lower level [24, 26]. According to these findings, light appears to up-regulate the F3’5’H pathway more than the F3’H pathway. However, our investigation did not reveal similar results. This might be because the rain-shelter application only caused a decrease in solar radiation inside the shelter at midday.

The volatile profile of grapes is mainly determined by variety, geographical origin, vintage, and climate conditions [27–29]. Light exposure affects the accumulation of volatiles in grapes in a complicated manner. For example, adequate light and appropriate shading of grape clusters were reported to favor the accumulation of volatiles in grape berries [30]. However, excessive exposure to solar radiation or over-covering of grapes was found to negatively affect the metabolism of volatiles [31, 32]. The present investigation showed that the formation of isoprenoid-derived volatiles was enhanced in the rain-shelter grapes. This indicated that the rain-shelter treatment in this study provided the grapes with a relatively moderate exposure to light, which might favor the metabolism of isoprenoid volatiles. It is also worth noting that the carbon allocation in the isoprenoid metabolism was predominately distributed to terpenoid synthesis, rather than norisoprenoid synthesis, in the rain-shelter-cultivated grapes. Accordingly, we hypothesized that this attenuated solar radiation caused by the rain shelter could help to promote the formation of terpene by triggering the adequate expression of terpene synthase [33, 34]. However, the effect of solar radiation on the terpene metabolism in grape berries is complicated. Bureau and his colleagues observed that compared with sun-exposed and naturally shaded cultivation, artificially shaded bunches resulted in lower levels of monoterpenols and C13 norisoprenoids in Muscat grapes (Muscat of Frontignan, *Vitis vinifera* L) [13]. By contrast, cluster-zone leaf removal treatments have been reported to increase the level of terpenoid compounds [35, 36]. Therefore, the effect of rain-shelter cultivation on terpene metabolism in grape berries might be related to the actual solar radiation intensity reaching the grape clusters. From the data obtained in this study, it was confirmed that the rain-shelter application helped to promote terpene biosynthesis in the isoterpene metabolism of Chardonnay grapes.

Phenylalanine is a key precursor for phenolic compounds and benzenoids, and it is produced in the shikimate pathway [10]. It has been demonstrated that supplemental UV radiation can induce the formation of flavonoids via up-regulation of relative gene expression under the shikimate pathway [37, 38]. However, lowering the UV radiation can reduce the production of phenylalanin-derived volatiles and flavonoids. In the present study, the rain-shelter treatment attenuated the solar and UV radiation inside the shelter at midday, which might be one of the most important factors that caused a decrease on the levels of the phenylalamin-derived volatiles and the total flavonoids in the rain-shelter-cultivated grapes.

Vintage appeared to have much greater impact on the accumulation of phenolic and volatile compounds compared with the cultivation treatment. The 2013 vintage season had a considerably higher sunshine duration during the grape developmental stages compared with the 2012 growing season, particularly during flowering to the end of veraison (stages I and II). The enhanced sunshine duration during the early stage of grape development led to a moderate elevation in the solar radiation that reached the grapes, which promoted the synthesis of phenolic compounds in the 2013 vintage grapes [14, 25]. In addition, the 81–97 DAF stage in the 2012 vintage was exposed to a longer sunshine duration compared with the 2013 vintage, which helped to enhance the accumulation of isoprenoid-derived volatiles in the 2012 vintage grapes because these volatiles are synthesized mainly during the late stage of grape development [39]. Furthermore, water control during the late stage of grape development is reported to have beneficial effects on volatile accumulation in grapes [40]. In the present study, a lower rainfall amount during the late developmental stage of the 2012 vintage, compared with the 2013 vintage, might account for the higher levels of most of the volatiles synthesized in the 2012 vintage grapes.

## Conclusion

In conclusion, the rain-shelter treatment significantly reduced the total solar radiation, photosynthetically active radiation, and UV radiation reaching the grape clusters in both vintage seasons. The rain-shelter application improved the flavan-3-ols levels during the early and middle stages of grape development, but resulted in a decrease at the late developmental stage. The metabolic pathways of flavonoids were changed in the rain-shelter-cultivated grapes. The levels of terpenes were significantly increased in the grapes under rain-shelter cultivation in both vintages. The 2012 vintage grapes under the rain-shelter treatment showed higher levels of fatty acid-derived volatiles during all grape development stages except for the harvest point. The 2012 vintage grapes under the rain-shelter treatment exhibited a higher level of free benzenoids but a lower level of bound benzenoids. However, similar results were not found in the 2013 vintage grapes. The PCA analysis indicated that compared with cultivation treatment, vintage had a much greater impact on the accumulation of phenolic and volatile compounds. In addition, the rain-shelter cultivation approach changed the allocation of carbon during grape development.

## Supporting Information

S1 Fig The sum of phenolic acids, flavonols, flavan-3-ols in grapes under rain-shelter and open-field cultivation. * represents significant differences in the concentrations of compounds between the two cultivation modes (*p*<0.05).
(TIF)

S2 Fig Comparison of total concentrations of volatile compounds from three pathways. * represents significant differences in the concentrations of compounds between the two cultivation modes (*p*<0.05).
(TIF)
