# Supplementary figures and images for: Rain-Shelter Cultivation Modifies Carbon Allocation in the Polyphenolic and Volatile Metabolism of Vitis vinifera L. Chardonnay Grapes
Source: PLoS One. 2016 May 24;11(5):e0156117. doi: 10.1371/journal.pone.0156117 (PMC4878772; doi:10.1371/journal.pone.0156117)

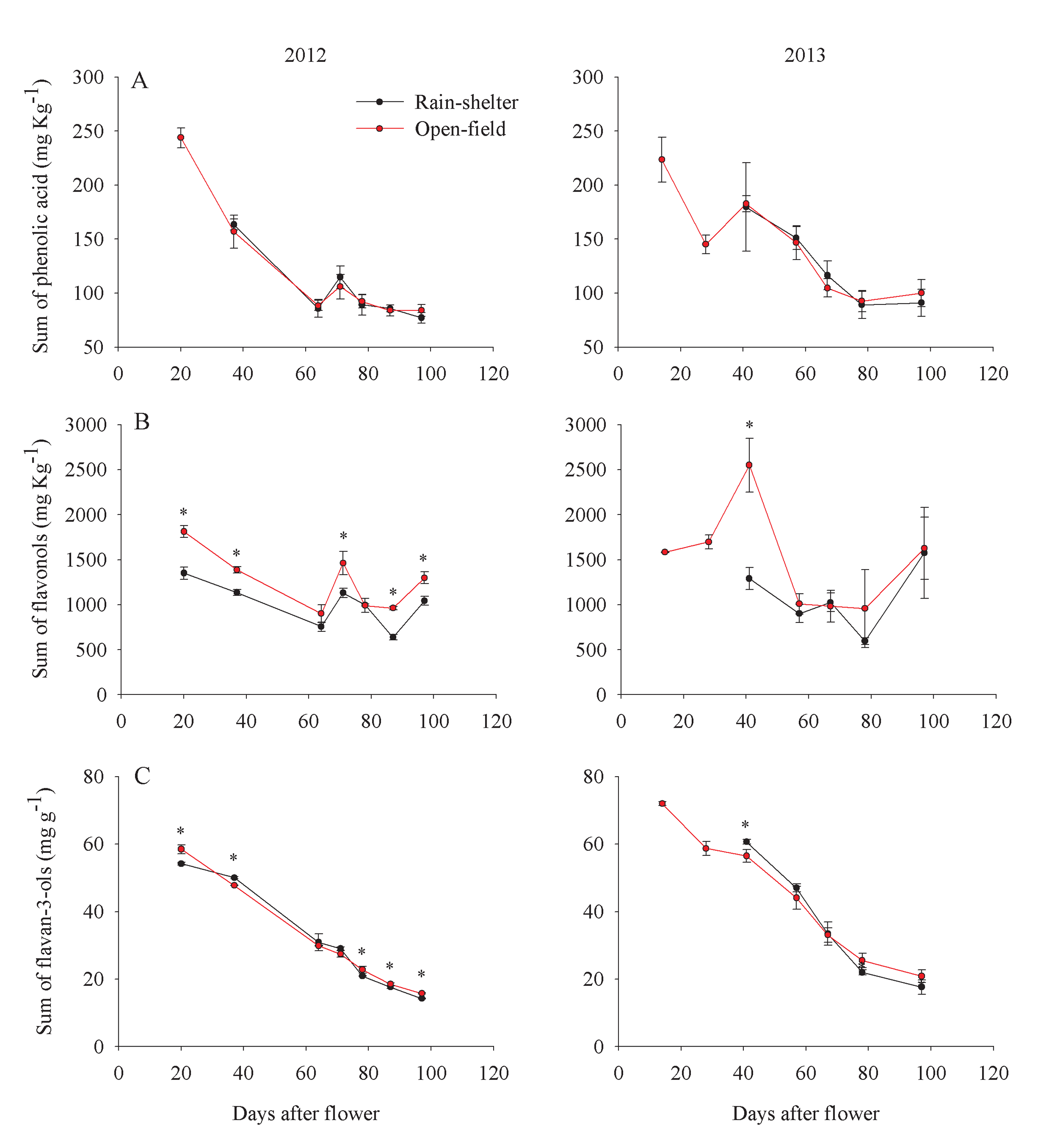

Supplement: S1 Fig — * represents significant differences in the concentrations of compounds between the two cultivation modes (p<0.05). (TIF) [file pone.0156117.s001.tif]

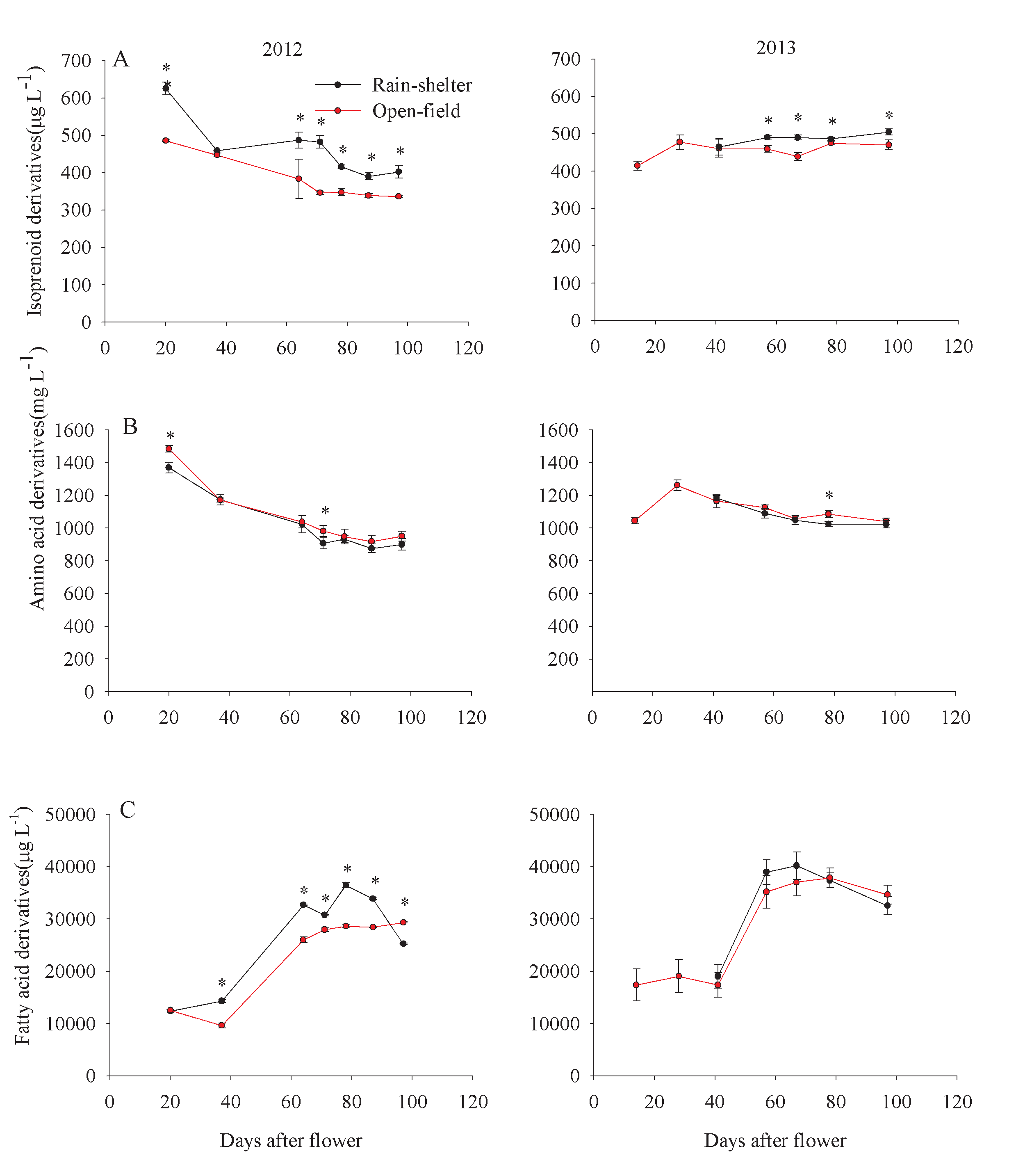

Supplement: S2 Fig — * represents significant differences in the concentrations of compounds between the two cultivation modes (p<0.05). (TIF) [file pone.0156117.s002.tif]
